# Phosphorylation of Maize Starch Enhanced with High-Voltage Electrical Discharge (HVED) Instead of Thermal Treatment

**DOI:** 10.3390/polym13193231

**Published:** 2021-09-23

**Authors:** Artur Gryszkin, Marijana Grec, Đurđica Ačkar, Tomasz Zięba, Antun Jozinović, Drago Šubarić, Borislav Miličević, Marijana Blažić, Jurislav Babić

**Affiliations:** 1 Department of Food Storage and Technology, Wrocław University of Environmental and Life Sciences, ul. Chełmońskiego, 37/41, 51-630 Wrocław, Poland; artur.gryszkin@upwr.edu.pl (A.G.); tomasz.zieba@upwr.edu.pl (T.Z.); 2 Faculty of Food Technology Osijek, Josip Juraj Strossmayer University of Osijek, Franje Kuhača 18, 31000 Osijek, Croatia; marijana.grec@ptfos.hr (M.G.); ajozinovic@ptfos.hr (A.J.); drago.subaric@ptfos.hr (D.Š.); bmilicevic@ptfos.hr (B.M.); jbabic@ptfos.hr (J.B.); 3 Polytechnic in Požega, Vukovarska 17, 34000 Požega, Croatia; 4 Department of Food Technology, Karlovac University of Applied Sciences, Trg Josipa Jurja Strossmayera 9, 47000 Karlovac, Croatia; mblazic@vuka.hr

**Keywords:** non-thermal process, Na_2_HPO_4_, Na_5_P_3_O_10_, rheological properties, viscoelastic properties

## Abstract

The aim of this research was to explore the use of a high-voltage electrical treatment (HVED) as a substitute for heating during the phosphorylation of maize starch. Starch was treated with HVED, phosphorylated with Na_2_HPO_4_ or Na_5_P_3_O_10_ with and without thermal treatment and phosphorylated in combination with HVED prior to and after the chemical modification. When starch was phosphorylated with Na_2_HPO_4_, HVED was more efficient in catalyzing reaction (3.89 mg P/kg for 30 min HVED in relation to 0.43 mg P/kg for thermal treatment), whereas with Na_5_P_3_O_10_ similar P content was achieved as with thermal treatment (0.76 P/kg for 30 min HVED in relation to 0.86 mg P/kg). The order of HVED and chemical reactions did not have a marked effect on phosphorous content. In combination with Na_2_HPO_4_, HVED pre-treatment had a more pronounced effect on the solubility and water absorption, whereas post-treatment was favoured with Na_5_P_3_O_10_. Mean diameter was increased by all treatments, where HVED had a marked effect. Enthalpy of gelatinization ranged from 11.76 J/g for starch treated with Na_5_P_3_O_10_ and 10 min-HVED to 13.58 J/g for Na_5_P_3_O_10_ treated sample. G′ and G″ increased after both thermally and HVED enhanced phosphorylations, with a slightly more pronounced effect of the HVED.

## 1. Introduction

Starch is found in numerous food products, from bread to yoghurt, where it has thickening, gelling or texture improving functions. Gelatinisation, rheological properties, water absorption and solubility of starches are important parameters for the evaluation of the effect of starch on food properties. Native starches often do not fulfil all the requirements for specific use in foods. Most often, they are not stable enough at high temperatures, in acidic conditions, during storage, or do not gelatinise at required temperatures and do not have desired rheological properties. In order to obtain the optimal effect and optimal aforementioned properties, starch has to be modified, which is most often done by chemical reactions [1].

Phosphorylation is a commonly applied modification procedure that requires a thermal treatment at high temperatures (130 °C up to 160 °C) in order to maximize the reaction efficiency. Generally, starch phosphates are prepared using sodium dihydrogenphosphate, disodium hydrogenphosphate [2], sodium tripolyphosphate and/or sodium trimetaphosphate. In all cases, both substituted and cross-linked starches may be obtained, depending on reaction conditions, and usually, a mixture of both products is produced. Ramadan and Sitohy [3] have recently reviewed mechanisms, properties and applications of phosphorylated starches in detail. They reported reaction times from 25 min to 180 min, with temperatures from 25 °C to 180 °C and phosphorus to starch ratio 0.0004–0.190. Phosphorylated starches generally have better freeze–thaw stability and larger content of resistant starch, along with altered gelatinisation and pasting properties in relation to native counterparts.

Since the process commonly requires high energy consumption and may result in undesired changes, such as colour degradation, deterioration of structure and formation of free radicals [3], a high-voltage electrical discharge (HVED) could be a good alternative, since it is a low-temperature process [4]. It is based on the application of short, high voltage pulses between two electrodes submerged in water. As a result of this, a photonic dissociation of water molecules leads to the liberation of OH˙ radicals and a large number of oxidising species, and UV light is emitted [5,6]. Shock waves are created which can physically damage the substrate, including starch granules. 

To this day, HVED has been applied as an extraction technique [7], decontamination method [8,9,10], wastewater treatment process [11] and different types of plasma have been used to modify starch [12]. To the best of our knowledge, HVED has not been used as a substitute for the thermal treatment during phosphorylation by other authors. 

The aim of this research is to examine if HVED may be used as an aid in phosphorylation of starch, instead of the thermal treatment, with phosphorus content and properties similar to the starch modified in classical manner. 

## 2. Materials and Methods

Native maize starch (C Gel™ max. 12% moisture; max. pH 5.5, max. SO_2_ 9.9; protein 0.5 g/100 g; fat 1 g/100 g; ash 0.1 g/100 g) was supplied by Cargill, Minneapolis, MN, USA. Na_2_HPO_4_ (p.a.) (CAS: 144-55-8) was supplied by T.T.T. d.o.o. Sveta Nedjelja, Croatia and Na_5_P_3_O_10_ (p.a.) (CAS: 7758-29-4) was from Acros Organics, Geel, Belgium.

### 2.1. Phosphorylation of Starch

#### 2.1.1. Phosphorylation with Na_2_HPO_4_

Modification with Na_2_HPO_4_ was conducted according to the method described by Prasanthi and Rama Rao [13] with slight modifications. Briefly, starch (100 g) and Na_2_HPO_4_ (30 g) were suspended in 200 mL of demineralised water with stirring at a magnetic stirrer for 30 min. The suspension was then centrifuged at 3000 rpm for 5 min (IEC Centra MP4R, International Equipment Company, Needham Heights, MA, USA), water was discarded, starch was washed three times with water (with centrifuging), and air-dried. 

For thermally treated phosphorylated starch, after the first centrifuging starch was air-dried overnight and thermally treated at 130 °C/2 h. Starch was then suspended in 250 mL of water, centrifuged, and washed three times with water. The obtained starch was dried at ambient temperature. 

#### 2.1.2. Phosphorylation with Na_5_P_3_O_10_

Phosphorylation with Na_5_P_3_O_10_ was done according to Lim and Seib [14] with and without thermal treatment at 130 °C. Briefly, 2.5 g of Na_5_P_3_O_10_ and 2.5 g of Na_2_SO_4_ was dissolved in 200 mL demineralized water. An amount of 100 g of starch was suspended in the solution by stirring at a magnetic stirrer for 20 min. pH of the suspension was set to 10.0 by drop-wise adding of NaOH (0.45 M) and the suspension was stirred for another hour. The suspension was centrifuged at 3000 rpm for 5 min (IEC Centra MP4R, International Equipment Company, Needham Heights, MA, USA), water was discarded and starch was washed three times with water, and air-dried.

For thermally treated phosphorylated starch, after centrifuging, starch was roasted in the oven at 130 °C/2 h, washed three times with water, and air-dried.

### 2.2. HVED Treatment of Starch

HVED device used in this research was custom-made by Ingeniare CPTS1, Osijek, Croatia and is described in more detail in Barišić et al. [15]. An amount of 200 g of starch was suspended in 200 mL of demineralized water and treated for 30 min with HVED (30 kV, 70 Hz) discharge from steel needle (diameter 2.5 mm) to plate electrode (diameter 45 mm), with 2 cm apart. During the whole time of the treatment, the suspension was stirred on a magnetic stirrer. After the HVED treatment, starch was centrifuged and air-dried until the desired moisture was reached or air-dried overnight, following by drying at 130 °C until the desired moisture.

### 2.3. Combination of HVED Treatment with Phosphorylation

Combined treatments were conducted by the HVED treatment for 10 or 30 min prior to the phosphorylation and vice-versa. For combined treatment, drying at 130 °C was omitted in order to explore if HVED may be used as a substituent for thermal reaction.

### 2.4. Determination of Phosphorus Content

The sample was prepared as described in PN-EN ISO 3946: 2000 and phosphorus was determined by ICP-OES.

In brief, the samples were digested "wet" in a closed microwave system with 5 cm^3^ of concentrated nitric acid (V) p.a. and 1 cm^3^ of concentrated hydrogen peroxide p.a., then the samples were mineralized in the microwave MARS 5 (CEM, Mathews, MC, USA) sample preparation system. The minerals were quantitatively transferred to 10 cm^3^ measuring vessels with redistilled water. An appropriate amount was taken for the determination of phosphorus. 

Phosphorus was determined by induced plasma atomic emission spectrometry—ICP-OES using the ICP-AES iCAP 7400 atomic emission spectrometer (Thermo Scientific, Waltham, MA, USA). The results were confirmed using the certified reference material NCS ZC 73012 - Cabbage, and the measurement uncertainty was estimated at 5%.

### 2.5. Determination of Swelling Power and Solubility in Water

Swelling power and solubility in water were determined at 80 °C, according to the method of Kapelko-Żeberska et al. [16]. A water suspension contained 1 g of starch per 100 g of the solution. The suspension was heated at 80 °C for 30 min with constant shaking. Afterwards, the sample was cooled to 20 °C and centrifuged for 30 min using a Biofuge 28RS centrifuge (Heraeus Sepatech, Hanau, Germany) with an acceleration of 22,500 *g*. The separated supernatant was used to determine dry matter content by the gravimetric method, and the precipitate left in centrifuge tubes was weighed.

### 2.6. Determination of the Mean Volumetric Diameter

Mean volumetric diameter was determined as described by Gryszkin et al. [17], after ultrasound-disintegration of starch agglomerates, using Masterizer 2000 (Malvern Instruments LTD, Malvern, UK) with Hydro 2000 MU adapter, at 20 °C and obscurantity between 15 and 20%. 

### 2.7. Determination of Thermal Characteristics 

Thermal properties were determined using the differential scanning calorimeter DSC 822E (MettlerToledo, Giessen, Germany), following methodology provided by Zieba, Szumny and Kapelko [18], with modification regarding measurement temperature range. Before the measurement, the calorimeter was calibrated using a sample of indium and a sample of zinc. The starch preparation (10 mg on a dry matter basis) was weighed into ME-51119871 medium-pressure crucibles, and bidistilled water was added at a ratio of 3:1 (water: starch). Afterwards, the crucible was sealed and conditioned at 25 °C for 30 min. The analysis was carried out in a temperature range of 25 °C–100 °C at a heating rate of 4 °C min^−1^. 

### 2.8. Rheological Measurements

#### 2.8.1. Determination of the Flow Curves of Pastes

The flow curves at 50 °C, with a shear rate range of 1–300 s^−1^, were determined according to Zieba, Juszczak and Gryszkin [19]. The 5% starch suspensions were heated at 96 °C for 30 min, with constant stirring. The hot paste was placed in a set of coaxial cylinders (Z38AL type) of the RS6000 HAKKE rheometer (Karlsruhe, Germany) then cooled and allowed to rest at 50 °C for 15 min. The flow curves were described by the power low (1), Herschel Bulkley (2), and Casson Equations (3):

(1) $$\mathrm{Power}\;\mathrm{low}\;\tau =K\;{\dot{\gamma }}^{n}$$

(2) $$\mathrm{Herschel}–\mathrm{Bulkley}\;\tau \;=\;\tau_{0}+K_{\mathrm{HB}}\;{\dot{\gamma }}^{n}$$

(3) $$\mathrm{Casson}\;\tau ={\tau_{0C}}^{0.25}+{\left(\eta_{C}\;\dot{\gamma }\right)}^{0.25}$$

where: n—flow behaviour index, $\dot{\gamma }$—shear rate (s^−1^), τ—shear stress (Pa), K—consistency coefficient (Pa∙s), K_HB_—consistency coefficient by Bulkley’s (Pa∙s), τ_0_—yield stress by Bulkley’s (Pa), τ_0C_—yield stress by Casson’s (Pa), η_C_—Casson’s plastic viscosity (Pa∙s).

#### 2.8.2. Determination of the Mechanical Spectra of Gels

The mechanical spectra of gels were obtained at 25 °C. The paste was obtained as described in the designation of the flow curves. The hot paste was placed in a measuring system (Z38AL type) of the RS6000 rheometer, then cooled and allowed to rest at 25 °C for 15 min. Measurements were taken in the linear viscoelastic range at a constant strain of 0.03 and a frequency from 0.1 to 10 Hz [20]. Then, the obtained mechanical spectra of the tested gels were read as the viscoelastic properties (counted by frequency 1Hz): G′ (storage modulus) and G″ (loss modulus) of native and modified maize starches. Additionally, loss tangent (as a dependency (G″/G′)) was calculated on this basis.

### 2.9. Statistical Analysis

All measurements were done in triplicates. Statistical analysis of results was performed by analysis of variance and Fischer’s LSD test at *p* < 0.05 using Statistica^®^ 13 and Microsoft Excell 2016.

## 3. Results and Discussion

It is well established that corn starch naturally contains small amounts of phosphorus, primarily linked to C-6 in amylopectin, with a smaller number that may be linked to C-2 and C-3 [21] and in the form of phospholipids. The present research confirms this since 0.144 g P/kg starch was determined in native maize starch (Table 1).

After the HVED treatment, the amount of phosphorus declined. This may be caused by the extraction in the water or the formation of complexes in which phosphorus would be “masked” during determination. Namely, Du et al. [22] reported that OH and O_2_ formed by HVED reacted with an aromatic ring, resulting in ring-cleavage products, and Grinevich et al. [11] reported a decrease in Pb, Cd and Mn in wastewater after HVED treatment, showing the influence of the treatment both on organic and inorganic compounds and different mechanisms of reactions.

Although phosphorylation with Na_2_HPO_4_ increased the amount of P, showing that the reaction occurred, it was not significantly efficient unless thermal treatment or HVED was applied, with the major advantage of HVED treatment over the thermal treatment (2.781–3.892 g P/kg compared to 0.434 g/kg). Interestingly, if HVED treatment lasted 10 min, the efficiency of the reaction was lower when HVED was applied after phosphorylation with Na_2_HPO_4_ (3.411 g/kg in relation to 3.831 g/kg when HVED was applied prior to phosphorylation). The opposite was observed for the 30 min-treatment, where higher content of P was determined when HVED was applied after the phosphorylation (3.892 g/kg in relation to 2.781 g/kg).

When phosphorylation was conducted with Na_5_P_3_O_10_, the reaction was not efficient at all unless thermal treatment or HVED were applied (Table 1). In this case, the HVED was somewhat less efficient than the thermal treatment and the difference considering duration and sequence was not so pronounced.

Malumba et al. [23] reported a partial gelatinization of starch during drying corn kernels at 130 °C, resulting in a higher swelling power. Since the same temperature was applied in this research, it may be concluded that the partial gelatinization of starch enabled better contact of the starch with phosphorylating agents, therefore enhancing the reactions. The more pronounced effect on reaction with Na_5_P_3_O_10_ supports this—without the thermal treatment, the reaction is negligible, while the thermally treated starch reacted with the reagent due to the better contact surface and probably penetration of the reagent into the granule.

The complexity of the results obtained for the combination of phosphorylation and HVED treatment may be explained by the complex effect of the HVED on starch. Namely, the release of energy into the water suspension induced by a high-voltage electrical discharge causes physical damage of starch granules, formation, and enlargement of fissures and pores. This facilitates the penetration of water and active compounds into the starch granule. Along with this, ionization and formation of free radicals and other active compounds in water occur, due to shearing and waves formed by HVED: H and OH radicals and ions, singlet O, O_2_, H_2_O_2_, etc. [24]. These compounds are in continuous contact with the starch and react with it, activating it for further reactions of depolymerization and cross-linking [25,26,27]. Depending on which one of them is predominant, starch will be more (depolymerization dominant over cross-linking) or less (cross-linking dominant over depolymerization) prone to react with phosphorylating agents. Since collisions and the combined effect of physical and chemical modifications of starch induced by HVED are random and hard to control, the exact mechanisms and conditions that will benefit one or the other reaction are yet to be revealed.

**Figure 1:**
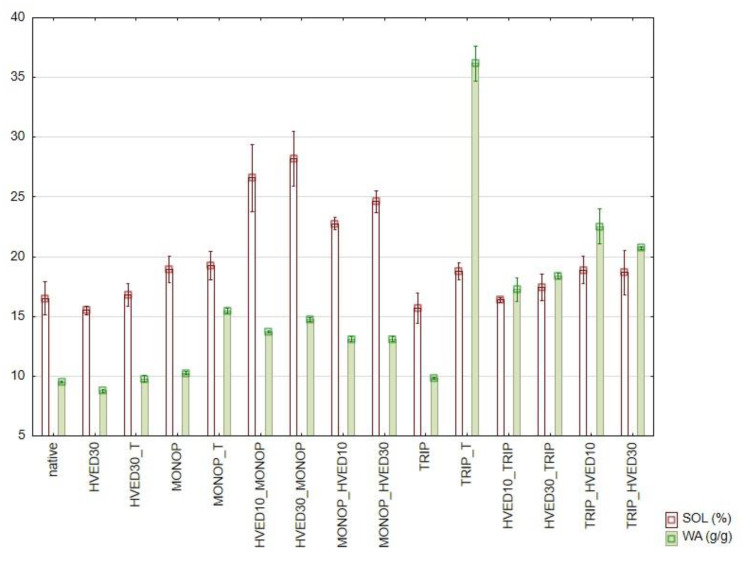
Solubility (SOL) and water absorption (WA) of native and modified maize starches (HVED10 = 10 min treatment with HVED; HVED30 = 30 min treatment with HVED; MONOP = Na_2_HPO_4_; TRIP = Na_5_P_3_O_10_; T = thermal treatment).

The chemical and physical modifications changed the affinity of starch towards the water. After the 30 min-HVED treatment, solubility and water absorption slightly decreased (Figure 1), indicating that the starch granules became more rigid and hydrophobic.

The same was observed by Andrade et al. [28] for starch films treated by low-pressure glow 1-butene plasma, although more often increase in swelling power and solubility is reported by authors, as shown in the review of Thirumdas, Kadam and Annapure [29]. The swelling power and the water absorption values of the starch phosphorylated with Na_2_HPO_4_ were higher than the native starch. As with P content, the thermal treatment enhanced the effect of modification on this property, and the HVED treatment was even more effective, with a more pronounced effect when applied prior to chemical modification. The modification with Na_5_P_3_O_10_ without an additional treatment did not have an effect on the swelling power (SP) and the solubility (SOL), confirming that the chemical reaction did not occur or was too moderate to result in the change of these properties. When the starch was heated after the reaction, the water absorption increased several times, and the swelling power increased, although not so markedly. Ascheri, Pereira and Bastos [30] also reported the increase in swelling and solubilization of starch due to phosphorylation. They attributed this to the fact that phosphate groups absorb large quantities of water, repel each other and facilitate penetration of water into the starch granule.

Again, the HVED treatment was not as effective as with Na_2_HPO_4_, although the increase in WA and SOL may be observed. Although the increase in swelling power and solubility of starch is primarily linked to the substitution of starch, Wongsagonsup et al. [31] claim that this effect is also observed at lower levels of cross-linking by phosphorylation, due to easier penetration of water and leaching of the starch into the solution. WA and SOL may be linked to the particle size shown in Table 2.

Namely, after all modifications, the average particle size increased due to the introduction of water molecules and phosphorylation.

Gelatinization parameters determined by DSC are shown in Table 2. Onset and conclusion temperatures of gelatinization decreased after the HVED treatment. The enthalpy of gelatinization increased indicating that more energy is required to disrupt starch granules and solubilize starch polymers, which is consistent with the observed decrease in water absorption and solubility. The phosphorylation also increased the enthalpy of gelatinization, indicating that cross-linking has occurred.

The HVED treatment combined with phosphorylation generally decreased the gelatinisation enthalpy, hence, the gelatinisation was easier than for phosphorylated starches. This is also consistent with the increased WA and SOL of dual-modified starches compared to phosphorylated ones. Ascheri, Pereira and Bastos [30] reported a decrease in gelatinisation enthalpy with the increase in phosphorus content in modified starches, however, this trend was not observed in the present research.

Rheological models are given in Table 3.

Three models were applied to describe rheological properties: Ostwald de Waele, Herschel Bulkly and Casson. As can be seen from the R^2^ values, all three models fit well, although the Ostwald de Waele has an R^2^ value as low as 0.96 for some samples.

The *n* value determined by the Ostwald de Waele model is between 0.359 and 0.483, showing pronounced non-Newtonian characteristics of all analysed starches. While the combination of HVED with Na_2_HPO_4_ resulted in lower values of *n* compared to the combination of phosphorylation with thermal treatment, when the phosphorylation was done with Na_5_P_3_O_10_, the shear-thinning effect was more pronounced when the thermal treatment was applied compared to combination with HVED. Both K and *n* values are close to the ones reported by Hornung et al. [32] for white yam starch. However, τ_0_ and K_HB_ in the present research are significantly larger than the ones reported by Hornung et al. [32]. This indicates a more pronounced plastic character of the pastes investigated in this research, and that a larger force must be applied in order to start the flowing, along with a higher initial viscosity. The combination of HVED with Na_2_HPO_4_ modification resulted in larger values of τ_0_ compared to the thermally treated counterpart (except the sample that was modified with Na_2_HPO_4_ with subsequent 10 min HVED treatment), i.e., higher stress must be applied to the HVED treated monophosphorylated starch in order to start it to flow. The opposite was observed for the Na_5_P_3_O_10_ modification: higher τ_0_ were observed in combination with the thermal treatment.

Casson model does not fully confirm the yield stress determined by the Herschel–Bulkley model, with τ_0C_ value of 10min-HVED treated Na_2_HPO_4_ modified starch lower than the thermally treated counterpart.

However, in both models, the difference between the values was not statistically significant so this discrepancy is not relevant. Casson’s plastic viscosity is the measure of the final viscosity of pastes (at the end of shearing). From the values in the Table 4, it is visible that the final viscosity follows the order: non-phosphorylated starches (η_C_ 0.044–0.047 Pas) < starches phosphorylated with Na_2_HPO_4_ (0.046–0.059 Pas) < starches phosphorylated with Na_5_P_3_O_10_ (0.056–0.086 Pas). Park, Chung and Yoo [33] ascribed higher viscosity to the associative effect of starch chains, while the shear thinning behaviour, they explained, “by breaking of an entangled polysaccharide molecule network during shearing”.

Storage modulus (G′), loss modulus (G″) and loss tangent (counted by frequency 1Hz) (G″/G′) are shown in Table 4.

G′ and G″ increased after both thermally induced and HVED enhanced phosphorylations, with a slightly more pronounced effect of the HVED compared to the thermal treatment. This is due to the formation of a strong gel network, according to Heo, Lee and Chang [34] and may be linked to the larger solubility and water absorption (Figure 1). However, there is no significant difference in G″/G′ values, which are mostly in the range 0.13 – 0.16, with the exception of phosphorylated starches without additional treatment (0.21 for Na_2_HPO_4_ and 0.26 for Na_5_P_3_O_10_). The values G″/G′ are close to 0.1 showing a formation of a gel network and the elastic nature of formed gels [34]. Although significant changes in G″/G′ values were not observed in this research, an HVED treatment may induce both decreases of these values due to cross-linking, or increases due to depolymerisation [33], and Heo, Lee and Chang [34] reported a decrease in G″/G′ values for a phosphorylated potato starch due to the cross-linking.

## 4. Conclusions

The results obtained in the present research show that an HVED might be used as a substitute for the thermal treatment in a phosphorylation reaction, with a similar or even better effect on the reaction efficiency, visible from both the P content and the investigated properties of starches. Starches with a similar P content were obtained when the HVED was applied instead of the thermal treatment in Na_5_P_3_O_10_ modification, and even better results were achieved in Na_2_HPO_4_ modification. Accordingly, the investigated properties (water absorption, solubility, rheological properties) were either similar to the thermally treated phosphorylated starches or improved in relation to them. Although additional research is needed to confirm the phenomena behind the HVED efficiency, it is presumably due to the activation of starch molecules both by energy increase and by polarization of active sites.

## Figures and Tables

**Table 1:** Phosphorus content in native and modified maize starches (HVED10 = 10 min treatment with HVED; HVED30 = 30 min treatment with HVED; MONOP = Na_2_HPO_4_; TRIP = Na_5_P_3_O_10_; T = thermal treatment).

Treatment	Phosphorus Content (g/kg)
native	0.144 ± 0.002^a^
HVED30	0.137 ± 0.003^a^
HVED30_T	0.139 ± 0.005^a^
MONOP	0.187 ± 0.003^a^
MONOP_T	0.434 ± 0.011^a,b^
HVED10_MONOP	3.831 ± 0.014^c^
HVED30_MONOP	3.411 ± 0.009^c^
MONOP_HVED10	2.781 ± 0.012^c^
MONOP_HVED30	3.892 ± 0.021^c^
TRIP	0.168 ± 0.009^a^
TRIP_T	0.855 ± 0.011^b^
HVED10_TRIP	0.749 ± 0.014^b^
HVED30_TRIP	0.628 ± 0.008^b^
TRIP_HVED10	0.706 ± 0.009^b^
TRIP_HVED30	0.757 ± 0.012^b^

Values with different superscripts are statistically different (*p* < 0.05).

**Table 2:** Gelatinization parameters and mean volumetric diameter of native and modified maize starches (HVED10 = 10 min treatment with HVED; HVED30 = 30 min treatment with HVED; MONOP = Na_2_HPO_4_; TRIP = Na_5_P_3_O_10_; T = thermal treatment).

Treatment	DSC Gelatinisation Parameters ^A^			Mean Volumetric Diameter (µm) ^B^
	t_o_ (°C) ^B^	t_c_ (°C) ^B^	ΔH (J/g) ^B^	
native	64.36 ± 0.04^f^	75.17 ± 0.02^f^	12.28 ± 0.37^b^	14.55 ± 0.01^a^
HVED30	64.17 ± 0.09^f^	74.79 ± 0.05^e^	12.90 ± 0.43^d–f^	21.10 ± 0.51^i^
HVED30_T	62.84 ± 0.04^c,d^	74.04 ± 0.08^a^	12.56 ± 0.11^b–e^	19.51 ± 0.03^h^
MONOP	65.23 ± 0.60^g^	75.00 ± 0.08^f^	13.27 ± 0.59^f–h^	17.58 ± 0.03^e^
MONOP_T	62.61 ± 0.04^b,c^	73.94 ± 0.16^a^	12.92 ± 0.05^d–f^	17.61 ± 0.02^e^
HVED10_MONOP	65.64 ± 0.10^h^	76.20 ± 0.08^j^	13.44 ± 0.35^g,h^	17.20 ± 0.13^d^
HVED30_MONOP	65.30 ± 0.19^g^	75.90 ± 0.01^h^	12.83 ± 0.01^c–f^	17.50 ± 0.03^e^
MONOP_HVED10	65.13 ± 0.01^g^	75.70 ± 0.26^g^	12.26 ± 0.18^b^	17.57 ± 0.02^e^
MONOP_HVED30	65.62 ± 0.12^h^	76.49 ± 0.11^i^	12.48 ± 0.07^b–d^	16.93 ± 0.02^c^
TRIP	64.24 ± 0.05^f^	74.74 ± 0.06^d,e^	13.58 ± 0.03^h^	16.67 ± 0.04^b^
TRIP_T	61.18 ± 0.08^a^	74.50 ± 0.01^b,c^	12.35 ± 0.22^b,c^	16.73 ± 0.01^b,c^
HVED10_TRIP	62.98 ± 0.10^d,e^	74.75 ± 0.04^d,e^	12.45 ± 0.50^b–d^	21.50 ± 0.13^j^
HVED30_TRIP	63.16 ± 0.03^e^	74.36 ± 0.07^b^	13.02 ± 0.17^e–g^	18.30 ± 0.03^g^
TRIP_HVED10	63.02 ± 0.25^d,e^	74.56 ± 0.25^c,d^	11.76 ± 0.33^a^	17.94 ± 0.03^f^
TRIP_HVED30	62.52 ± 0.03^b^	74.52 ± 0.05^b,c^	13.02 ± 0.06^e–g^	21.10 ± 0.17^i^

^A^ t_o_, onset temperature; t_c_, conclusion temperature; ΔH, gelatinization enthalpy. ^B^ Values with different superscripts in the same column are statistically different (*p* < 0.05).

**Table 3:** Rheological properties of native and modified maize starches described with different models (HVED10 = 10 min treatment with HVED; HVED30 = 30 min treatment with HVED; MONOP = Na_2_H_P_O_4_; TRIP = Na_5_P_3_O_10_; T = thermal treatment).

Ostwald de Waele			
Treatment	K (Pas^n^)	n	R^2^
native	2.331 ± 0.052^a^	0.483 ± 0.003^d–f^	0.9878
HVED30	3.151 ± 0.383^a^	0.450 ± 0.012^c^	0.9823
HVED30_T	2.492 ± 0.105^a^	0.468 ± 0.007^f,g^	0.9819
MONOP	3.261 ± 0.657^a^	0.453 ± 0.033^c^	0.9947
MONOP_T	5.538 ± 0.577^b,c^	0.411 ± 0.014^g^	0.9797
HVED10_MONOP	5.404 ± 1.857^b^	0.410 ± 0.061^a,b^	0.9745
HVED30_MONOP	6.572 ± 1.054^b–d^	0.369 ± 0.021^c–e^	0.9699
MONOP_HVED10	5.297 ± 0.521^b^	0.403 ± 0.011^a^	0.9784
MONOP_HVED30	6.828 ± 1.156^c,d^	0.359 ± 0.023^b,c^	0.9648
TRIP	2.623 ± 0.135^a^	0.488 ± 0.013^a,b^	0.9869
TRIP_T	11.737 ± 1.181^e^	0.371 ± 0.013^f,g^	0.9718
HVED10_TRIP	7.094 ± 0.584^d^	0.421 ± 0.007^c–e^	0.9870
HVED30_TRIP	6.565 ± 0.902^b–d^	0.426 ± 0.025^b,c^	0.9888
TRIP_HVED10	7.798 ± 0.412^d^	0.401 ± 0.013^c,d^	0.9839
TRIP_HVED30	7.672 ± 0.606^d^	0.415 ± 0.011^e,f^	0.9873
**Herschel Bulkly**			
**Treatment**	**τ_0_ (Pa)**	**K_HB_ (Pas^n^)**	**R^2^**
native	8.057 ± 0.197^a-c^	0.335 ± 0.007^a,b^	0.9990
HVED30	10.586 ± 1.091^c^	0.314 ± 0.018^a,b^	0.9980
HVED30_T	9.083 ± 0.380^b,c^	0.236 ± 0.015^a^	0.9975
MONOP	7.106 ± 1.238^a,b^	0.977 ± 0.073^e^	0.9996
MONOP_T	15.867 ± 1.505^d,e^	0.574 ± 0.035^c,d^	0.9960
HVED10_MONOP	16.250 ± 4.444^d,e^	0.370 ± 0.105^a,b^	0.9967
HVED30_MONOP	18.017 ± 2.209^d-f^	0.368 ± 0.044^a,b^	0.9942
MONOP_HVED10	15.123 ± 1.667^d^	0.481 ± 0.064^b,c^	0.9959
MONOP_HVED30	18.850 ± 2.494^e,f^	0.290 ± 0.055^a^	0.9931
TRIP	5.178 ± 0.532^a^	1.101 ± 0.182^e,f^	0.9998
TRIP_T	32.323 ± 2.776^g^	0.711 ± 0.107^d^	0.9982
HVED10_TRIP	18.650 ± 1.530^e,f^	1.133 ± 0.039^e,f^	0.9986
HVED30_TRIP	16.737 ± 2.867^d,e^	1.187 ± 0.199^f^	0.9991
TRIP_HVED10	20.517 ± 1.287^f^	0.970 ± 0.155^e^	0.9985
TRIP_HVED30	19.697 ± 2.011^f^	1.224 ± 0.139^f^	0.9988
**Casson**			
**Treatment**	**τ_0C_ (Pa)**	**η_C_ (Pas)**	**R^2^**
native	5.805 ± 0.111^a^	0.047 ± 0.000^a-c^	0.9985
HVED30	7.489 ± 0.779^a^	0.047 ± 0.002^a-c^	0.9969
HVED30_T	6.080 ± 0.201^a^	0.044 ± 0.001^a^	0.9962
MONOP	7.599 ± 1.213^a^	0.050 ± 0.005^b-d^	0.9990
MONOP_T	12.337 ± 1.024^b^	0.059 ± 0.002^f^	0.9952
HVED10_MONOP	11.755 ±3.123^b^	0.055 ± 0.009^d,e^	0.9945
HVED30_MONOP	13.477 ± 1.599^b^	0.048 ± 0.002^a-c^	0.9924
MONOP_HVED10	11.590 ± 0.941^b^	0.053 ± 0.001^c–e^	0.9951
MONOP_HVED30	13.740 ± 1.695^b,c^	0.046 ± 0.002^a,b^	0.9903
TRIP	6.425 ± 0.217^a^	0.056 ± 0.003^d,e^	0.9985
TRIP_T	24.443 ± 1.825^e^	0.086 ± 0.002^g^	0.9960
HVED10_TRIP	16.080 ± 1.156^d^	0.083 ± 0.003^g^	0.9982
HVED30_TRIP	14.907 ± 1.597^c,d^	0.081 ± 0.007^f,g^	0.9987
TRIP_HVED10	17.067 ± 0.570^d^	0.076 ± 0.005^f^	0.9980
TRIP_HVED30	17.170 ± 1.065^d^	0.085 ± 0.002^g^	0.9984

Values with different superscripts are statistically different (*p* < 0.05).

**Table 4:** Viscoelastic properties (storage modulus (G′), loss modulus (G″) and loss tangent (counted by frequency 1Hz) (G″/G′)) of native and modified maize starches (HVED10 = 10 min treatment with HVED; HVED30 = 30 min treatment with HVED; MONOP = Na_2_HPO_4_; TRIP = Na_5_P_3_O_10_; T = thermal treatment).

Treatment	G′ (Pa)	G″ (Pa)	G″/G′
native	33.90 ± 0.69^a,b^	4.64 ± 0.04^b^	0.14 ± 0.00^a^
HVED30	40.04 ± 3.14^b,c^	5.23 ± 0.36^c^	0.13 ± 0.00^a^
HVED30_T	31.49 ± 2.06^a^	4.05 ± 0.10^a^	0.13 ± 0.01^a^
MONOP	29.23 ± 9.60^a^	5.83 ± 0.29^d^	0.21 ± 0.07^b^
MONOP_T	41.52 ± 2.41^b,c^	6.62 ± 0.20^e^	0.16 ± 0.00^a^
HVED10_MONOP	45.66 ± 0.78^c^	6.14 ± 0.18^d,e^	0.13 ± 0.01^a^
HVED30_MONOP	43.04 ± 2.39^c^	6.00 ± 0.14^d^	0.14 ± 0.01^a^
MONOP_HVED10	41.14 ± 2.09^b,c^	5.99 ± 0.20^d^	0.15 ± 0.00^a^
MONOP_HVED30	43.77 ± 3.16^c^	5.91 ± 0.14^d^	0.14 ± 0.01^a^
TRIP	26.56 ± 7.68^a^	6.00 ± 0.26^d^	0.24 ± 0.05^b^
TRIP_T	57.45 ± 1.06^d,e^	9.03 ± 0.21^f^	0.16 ± 0.00^a^
HVED10_TRIP	69.27 ± 1.26^f^	9.75 ± 0.10^g^	0.14 ± 0.00^a^
HVED30_TRIP	61.90 ± 10.88^d-f^	8.99 ± 0.80^f^	0.15 ± 0.01^a^
TRIP_HVED10	56.92 ± 3.66^d^	8.79 ± 0.49^f^	0.15 ± 0.00^a^
TRIP_HVED30	64.52 ± 1.96^e,f^	9.62 ± 0.12^g^	0.15 ± 0.00^a^

Values with different superscripts are statistically different (*p* < 0.05).

## Data Availability

The data presented in this study are available on request from the corresponding author.
